# Supplementary material for: Hybrid Models and Biological Model Reduction with PyDSTool
Source: PLoS Comput Biol. 2012 Aug 9;8(8):e1002628. doi: 10.1371/journal.pcbi.1002628 (PMC3415397; doi:10.1371/journal.pcbi.1002628)
Supplement: Text S4 — Complete source code for the PyDSTool package (version 0.88.120504). Includes API documentation and help files linking to web pages. This file is identical to the current public release on Sourceforge.net. (ZIP) [file pcbi.1002628.s004.zip › PyDSTool/html/PyDSTool.Toolbox.ParamEst-pysrc.html]

xml version="1.0" encoding="ascii"?


PyDSTool.Toolbox.ParamEst


| Home | Trees | Indices | Help | PyDSTool |
| --- | --- | --- | --- | --- |

Package PyDSTool ::
Package Toolbox ::
Module ParamEst

[hide private]

[frames] | no frames]

# Source Code for Module PyDSTool.Toolbox.ParamEst

```
   1  """Parameter estimation classes for ODEs. 
   2   
   3     Robert Clewley. 
   4  """ 
   5   
   6  from __future__ import division 
   7   
   8  # PyDSTool imports 
   9  from PyDSTool.Points import Point, Pointset 
  10  from PyDSTool.Model import Model 
  11  from PyDSTool.common import Utility, _seq_types, metric, args, sortedDictValues, \ 
  12       remain, metric_L2, metric_L2_1D, metric_float, metric_float_1D 
  13  from PyDSTool.utils import intersect, filteredDict 
  14  from PyDSTool.errors import * 
  15  from PyDSTool import common 
  16  from PyDSTool.MProject import qt_feature_leaf, process_raw_residual 
  17  import PyDSTool.Redirector as redirc 
  18  from PyDSTool.matplotlib_import import pylab 
  19  from PyDSTool.Toolbox.optimizers import * 
  20   
  21  try: 
  22      from constraint import Problem, FunctionConstraint, RecursiveBacktrackingSolver, \ 
  23           BacktrackingSolver, MinConflictsSolver 
  24  except ImportError: 
  25      # constraint package must be installed to use some parameter estimation features: 
  26      # http://labix.org/python-constraint 
  27      Problem = None 
  28      FunctionConstraint = None 
  29      RecursiveBacktrackingSolver = None 
  30      BacktrackingSolver = None 
  31      MinConflictsSolver = None 
  32   
  33  from scipy.optimize import minpack, optimize 
  34  from numpy.linalg import norm, eig, eigvals, svd 
  35  from scipy.linalg import svdvals 
  36  from scipy.io import * 
  37  import sys, traceback 
  38  import operator 
  39   
  40  from numpy import linspace, array, arange, zeros, sum, power, \ 
  41       swapaxes, asarray, ones, alltrue, concatenate, rank, ravel, argmax, \ 
  42       argmin, argsort, float, sign 
  43  import numpy as np 
  44   
  45  import math, types 
  46  from copy import copy, deepcopy 
  47   
  48  try: 
  49      # use psyco JIT byte-compiler optimization, if available 
  50      import psyco 
  51      HAVE_PSYCO = True 
  52  except ImportError: 
  53      HAVE_PSYCO = False 
  54   
  55  # ------------------------------------------------------------------------ 
  56   
  57  _pest_classes = ['ParamEst', 'LMpest', 'BoundMin', 'residual_fn_context', 
  58                   'residual_fn_context_1D', 'L2_feature', 'L2_feature_1D'] 
  59   
  60  _deprecated_functions = ['get_slope_info', 'get_extrema', 
  61             'compare_data_from_events', 'get_extrema_from_events'] 
  62   
  63  _ctn_functions = ['do_2Dstep', 'do_2Ddirn', 'ctn_residual_info'] 
  64   
  65  _generic_opt = ['make_opt', 'restrict_opt'] 
  66   
  67  _utils = ['sweep1D', 'filter_feats', 'filter_pars', 'select_pars_for_features', 
  68            'grad_from_psens', 'norm_D_sum', 'filter_iface', 'organize_feature_sens'] 
  69   
  70  _errors = ['Converged', 'ConstraintFail'] 
  71   
  72  __all__ = _pest_classes + _deprecated_functions + _ctn_functions + \ 
  73          _generic_opt + _utils + _errors 
  74   
  75   


76 -class Converged(PyDSTool_Error):


77      pass

  78   


79 -class ConstraintFail(PyDSTool_Error):


80      pass

  81   
  82   
  83  solver_lookup = {'RecursiveBacktrackingSolver': RecursiveBacktrackingSolver, 
  84             'BacktrackingSolver': BacktrackingSolver, 
  85             'MinConflictsSolver': MinConflictsSolver} 
  86   
  87  # ---- 
  88  # Used to suppress output from legacy codes 
  89   
  90  rout = redirc.Redirector(redirc.STDOUT) 
  91  rerr = redirc.Redirector(redirc.STDERR) 
  92   
  93  # ---------------------------------------------------------------------------- 
  94   
  95  # Simple parameter continuation of residual vector (maintain residual constant 
  96  # while varying free parameters) -- assumes locally 'regular' landscape, esp. 
  97  # no folds, low curvature 
  98   


99 -def do_2Dstep(fun, p, dirn, maxsteps, stepsize, atol, i0, orig_res, orig_dirn, 
 100                all_records):


101      """ 
 102      Residual vector continuation step in 2D parameter space. 
 103   
 104      orig_dirn corresponds to direction of positive dirn, in case when 
 105      re-calculating gradient the sign flips""" 
 106      record = {} 
 107      print "Recalculating gradient" 
 108      grad = fun.gradient(p) 
 109      neut = np.array([grad[1], -grad[0]]) 
 110      neut = neut/norm(neut) 
 111      if np.sign(dot(neut, orig_dirn)) != 1: 
 112          print "(neut was flipped for consistency with direction)" 
 113          neut = -neut 
 114      print "Neutral direction:", neut 
 115      record['grad'] = grad 
 116      record['neut'] = neut 
 117      residuals = [] 
 118      # inner loop - assumes curvature will be low (no adaptive step size) 
 119      print "\n****** INNER LOOP" 
 120      new_pars = copy(p) 
 121      i = 0 
 122      while True: 
 123          if i > maxsteps: 
 124              break 
 125          new_pars += dirn*stepsize*neut 
 126          res = fun(new_pars) 
 127          d = abs(res-orig_res) 
 128          if res > 100 or d > atol: 
 129              # fail 
 130              break 
 131          step_ok = d < atol/2. 
 132          if step_ok: 
 133              r = (copy(new_pars), res) 
 134              residuals.append(r) 
 135              all_records[i0+dirn*i] = r 
 136              num_dirn_steps = len([k for k in all_records.keys() if \ 
 137                                    k*dirn >= abs(i0)]) 
 138              i += 1 
 139              print len(all_records), "total steps taken, ", num_dirn_steps, \ 
 140                      "in since grad re-calc: pars =", new_pars, " res=", res 
 141          else: 
 142              # re-calc gradient 
 143              break 
 144      if len(residuals) > 0: 
 145          record['p_new'] = residuals[-1][0] 
 146          record['n'] = i 
 147          record['i0_new'] = i0+dirn*i 
 148      else: 
 149          record['p_new'] = p 
 150          record['n'] = 0 
 151          record['i0_new'] = i0 
 152      return record

 153   
 154   


155 -def do_2Ddirn(fun, p0, dirn, maxsteps, stepsize, atol, orig_res, orig_dirn, 
 156                all_records):


157      """ 
 158      Residual vector continuation in a single neutral direction in 2D parameter 
 159      space, given by dirn = +1/-1 from point p0. 
 160   
 161      maxsteps is *per* direction""" 
 162      print "\nStarting direction:", dirn 
 163      dirn_rec = [] 
 164      p = p0 
 165      if dirn == 1: 
 166          # will count 0, 1, ... 
 167          i = 0 
 168      else: 
 169          # will count -1, -2, ... 
 170          i = -1 
 171      done = False 
 172      while not done: 
 173          print "Steps from i =", i 
 174          r = do_step(fun, p, dirn, maxsteps, stepsize, atol, i, orig_res, 
 175                      orig_dirn, all_records) 
 176          if r['n'] == 0: 
 177              # no steps taken successfully 
 178              done = True 
 179          else: 
 180              dirn_rec.append(r) 
 181              p = r['p_new'] 
 182              i = r['i0_new'] 
 183          if abs(i) > maxsteps: 
 184              done = True 
 185      return dirn_rec

 186   
 187   


188 -def ctn_residual_info(recs, do_plot=False):


189      """Temporary helper function for use with continuation functions.""" 
 190      ixlims = [min(recs.keys()), max(recs.keys())] 
 191      if do_plot: 
 192          pylab.figure() 
 193      r = [] 
 194      dr = [] 
 195      old_res = None 
 196      for i in range(ixlims[0], ixlims[1]+1): 
 197          pars, res = recs[i] 
 198          r.append(res) 
 199          if old_res is not None: 
 200              dr.append(-np.log(abs(res-old_res)/old_res)) 
 201          old_res = res 
 202          if do_plot: 
 203              pylab.plot(pars[0],pars[1],'ko') 
 204      return r, dr

 205   
 206   
 207   


208 -def sweep1D(fun, interval, resolution):


209      numpoints = (interval[1]-interval[0])/resolution + 1 
 210      ps = linspace(interval[0], interval[1], numpoints) 
 211      res = [] 
 212      for p in ps: 
 213          res.append(fun(array([p]))) 
 214      return ps, array(res)

 215   
 216   


217 -class residual_fn_context(helpers.ForwardFiniteDifferencesCache):


218 -    def _res_fn(self, p, extra_args=None):


219          # p comes in as an array 
 220          pest = self.pest 
 221          for i, parname in enumerate(pest.freeParNames): 
 222              pest.modelArgs[pest.parTypeStr[i]][parname] = p[i] 
 223          pest.testModel.set(**pest.modelArgs) 
 224          try: 
 225              return pest.evaluate() 
 226          except KeyboardInterrupt: 
 227              raise 
 228          except: 
 229              exceptionType, exceptionValue, exceptionTraceback = sys.exc_info() 
 230              print "******************************************" 
 231              print "Problem evaluating residual function" 
 232              print "  ", exceptionType, exceptionValue 
 233              for line in traceback.format_exc().splitlines()[-4:-1]: 
 234                  print "   " + line 
 235              print "  originally on line:", traceback.tb_lineno(exceptionTraceback) 
 236              if self.pest.verbose_level > 1: 
 237                  raise 
 238              else: 
 239                  print "(Proceeding with penalty values)\n" 
 240              return 10*ones(pest.context.res_len)

 241   


242 -class residual_fn_context_1D(helpers.ForwardFiniteDifferencesCache):


243 -    def _res_fn(self, p, extra_args=None):


244          # p comes in as an array 
 245          pest = self.pest 
 246          pest.modelArgs[pest.parTypeStr][pest.freeParNames[0]] = p 
 247          pest.testModel.set(**pest.modelArgs) 
 248          try: 
 249              return pest.evaluate()[0] 
 250          except KeyboardInterrupt: 
 251              raise 
 252          except: 
 253              exceptionType, exceptionValue, exceptionTraceback = sys.exc_info() 
 254              print "******************************************" 
 255              print "Problem evaluating residual function" 
 256              print "  ", exceptionType, exceptionValue 
 257              for line in traceback.format_exc().splitlines()[-4:-1]: 
 258                  print "   " + line 
 259              print "  originally on line:", traceback.tb_lineno(exceptionTraceback) 
 260              if self.pest.verbose_level > 1: 
 261                  raise 
 262              else: 
 263                  print "(Proceeding with penalty value)\n" 
 264              return 100

 265   
 266   
 267  # ---------------------------------------------------------------------------- 
 268   
 269  ## EXPERIMENTAL FUNCTIONS (in development) 
 270   
 271   


272 -def grad_from_psens(psens, pest):


273      pd2a = pest.pars_dict_to_array 
 274      pd = {} 
 275      for pname, fsens_dict in psens.items(): 
 276          res_list = [] 
 277          for feat, sens_array in fsens_dict.items(): 
 278              res_list.extend(list(sens_array)) 
 279          pd[pname] = sum() 
 280      return pd2a(pd)

 281   
 282   


283 -def filter_feats(parname, feat_sens):


284      """Filter features whose residual vectors show a *net* increase (dirn=1) 
 285      or decrease (dirn=-1) as one parameter is varied. Provided the 
 286      sensitivities were measured appropriately, dirn=0 will select any 
 287      non-smoothly changing features (e.g. discrete-valued). 
 288   
 289      feat_sens is a dictionary of feature sensitivities keyed by parameter 
 290      name, e.g. as returned by the ParamEst.par_sensitivity method. 
 291   
 292      Returns a list of ((model interface, feature), sensitivity) pairs, where 
 293      the feature belongs to the model interface (in case of duplication in 
 294      multiple interfaces), and the sensitivity is the absolute value of the 
 295      net increase/decrease. The lists are ordered by decreasing 
 296      magnitude of sensitivity. 
 297   
 298      Definition of net increase: 
 299      e.g. if sensitivity for a given feature with a 3-vector residual is 
 300      [-0.1 0.4 1.5] then the sum is +1.8 and will be selected for the 
 301      'increasing' direction. 
 302      """ 
 303      incr = [] 
 304      decr = [] 
 305      neut = [] 
 306      for mi, fdict in feat_sens[parname].iteritems(): 
 307          for f, sens in fdict.iteritems(): 
 308              sum_sens = sum(sens) 
 309              sign_ss = np.sign(sum_sens) 
 310              abs_ss = abs(sum_sens) 
 311              if alltrue(sign_ss == 1): 
 312                  incr.append(((mi,f), abs_ss, parname)) 
 313              elif alltrue(sign_ss == -1): 
 314                  decr.append(((mi, f), abs_ss, parname)) 
 315              else: 
 316                  neut.append(((mi, f), 0.)) 
 317      return sorted(incr, reverse=True, key=operator.itemgetter(1)), \ 
 318             sorted(decr, reverse=True, key=operator.itemgetter(1)), neut

 319   
 320   


321 -def filter_pars(mi_feat, feat_sens):


322      """ 
 323      For a given (model interface, feature) pair, find all parameters 
 324      that change the feature's net residual in the same direction, or not at all. 
 325      (Provided the sensitivities were measured appropriately, this will select 
 326      any non-smoothly changing features (e.g. discrete-valued)). 
 327   
 328      feat_sens is a dictionary of feature sensitivities keyed by parameter 
 329      name, e.g. as returned by the ParamEst.par_sensitivity method. 
 330   
 331      Returns a triple of lists of (parameter names, sensitivity) pairs: 
 332      increasing, decreasing, and neutral. The lists are ordered by decreasing 
 333      magnitude of sensitivity. 
 334      """ 
 335      mi, feat = mi_feat 
 336      incr = [] 
 337      decr = [] 
 338      neut = [] 
 339      for pname, fdict in feat_sens.iteritems(): 
 340          sens = fdict[mi][feat] 
 341          sum_sens = sum(sens) 
 342          sign_ss = np.sign(sum_sens) 
 343          abs_ss = abs(sum_sens) 
 344          if alltrue(sign_ss == 1): 
 345              incr.append((pname, abs_ss, (mi, feat))) 
 346          elif alltrue(sign_ss == -1): 
 347              decr.append((pname, abs_ss, (mi, feat))) 
 348          else: 
 349              neut.append((pname, 0., (mi, feat))) 
 350      return sorted(incr, reverse=True, key=operator.itemgetter(1)), \ 
 351             sorted(decr, reverse=True, key=operator.itemgetter(1)), neut

 352   
 353   


354 -def _present_and_sensitive(xsf, L, thresh, rejected, neutral):


355      """Helper function to return whether x is present in list L with an 
 356      associated sensitivity s larger than thresh, where L is made up of 
 357      (y, ysens) pairs, provided the sensitivities are larger than threshold. 
 358   
 359      xsf is the triple (x, xsens, feature). rejected and neutral arguments 
 360      should be lists to store the new rejected and neutral items. 
 361      """ 
 362      x, xs, xf = xsf 
 363      flag = None 
 364      p_and_s = False 
 365      for i, (y, ys, yf) in enumerate(L): 
 366          if x == y: 
 367              # present 
 368              x_is_sens = xs >= thresh 
 369              y_is_sens = ys >= thresh 
 370              if x_is_sens and y_is_sens: 
 371                  # clash 
 372                  p_and_s = True 
 373                  flag = i 
 374                  if xs > ys: 
 375                      rejected.append((x, xs, xf)) 
 376                  else: 
 377                      rejected.append((y, ys, yf)) 
 378              elif x_is_sens and not y_is_sens: 
 379                  # effecively not present in L, so delete 
 380                  p_and_s = False 
 381                  flag = i 
 382              elif not x_is_sens and y_is_sens: 
 383                  # effectively present in L 
 384                  p_and_s = True 
 385              else: 
 386                  # not x_is_sens and not y_is_sens 
 387                  # ... effectively not present in L but x too small to be 
 388                  # considered present in result anyway, so treat as p_and_s = True 
 389                  # (will be considered neutral) 
 390                  p_and_s = True 
 391                  flag = i 
 392                  if xs > ys: 
 393                      neutral.append((x, xs, xf)) 
 394                  else: 
 395                      neutral.append((y, ys, yf)) 
 396              break 
 397      if flag is not None: 
 398          # filter out item so that it's not kept in L 
 399          del L[i] 
 400      return p_and_s

 401   
 402   


403 -def _makeUnique(L):


404      seen = {} 
 405      for x, s, f in L: 
 406          if x in seen: 
 407              if s > seen[x][0]: 
 408                  seen[x] = (s, f) 
 409          else: 
 410              seen[x] = (s, f) 
 411      Lu = [(x,pair[0], pair[1]) for x, pair in seen.items()] 
 412      return sorted(Lu, reverse=True, key=operator.itemgetter(1))

 413   


414 -def pp(l):


415      """List pretty printer""" 
 416      print "[", 
 417      for x in l: 
 418          print x, "," 
 419      print "]"

 420   
 421   
 422  # --------------------------------- 
 423   
 424   


425 -def select_pars_for_features(desired_feats, feat_sens, deltas, neg_tol=0, pos_tol=0.001, 
 426                               method='RecursiveBacktrackingSolver', 
 427                               forwardCheck=True, verbose=False):


428      """Default tol > 0 in case there are undifferentiable features that will 
 429      never lead to satisfaction of constraints with tol=0. 
 430   
 431      Returns a problem object and the by-parameter sensitivity dictionary of 
 432      derivatives, D.""" 
 433      try: 
 434          solver = solver_lookup[method] 
 435      except KeyError: 
 436          print "Specified solver not found. Will try recursive backtracking solver" 
 437          solver = RecursiveBacktrackingSolver 
 438      try: 
 439          problem = Problem(solver(forwardCheck)) 
 440      except TypeError: 
 441          print "Install constraint package from http://labix.org/python-constraint" 
 442          raise ImportError("Must have constraint package installed to use this feature") 
 443      global D, feat_name_lookup, pars, par_deltas 
 444      par_deltas = deltas 
 445      pars = feat_sens.keys() 
 446      pars.sort() 
 447      num_pars = len(pars) 
 448      assert len(par_deltas) == num_pars 
 449   
 450      D = {} 
 451   
 452      max_val = 0 
 453      for par in pars: 
 454          for mi, fdict in feat_sens[par].iteritems(): 
 455              for f, ra in fdict.iteritems(): 
 456                  val = sum(ra) 
 457                  if norm(val) > max_val: 
 458                      max_val = val 
 459                  if f not in D: 
 460                      D[f] = {} 
 461                  D[f][par] = val 
 462   
 463   
 464      values = [-1, 0, 1] 
 465      problem.addVariables(pars, values) 
 466   
 467      feat_name_lookup = {} 
 468      for f in D.keys(): 
 469          feat_name_lookup[f.name] = f 
 470   
 471      constraint_funcs = [] 
 472      for f in D.keys(): 
 473          if f in desired_feats: 
 474              op = 'operator.le' 
 475              this_tol = neg_tol 
 476          else: 
 477              op = 'operator.lt' 
 478              this_tol = pos_tol 
 479          code =  "def D%s(*p):\n"%f.name 
 480          if verbose: 
 481              code += "  print '%s', p\n"%f.name 
 482          code += "  pd = D[feat_name_lookup['%s']]\n"%f.name 
 483          code += "  prods = [pd[par]*par_deltas[pi]*p[pi] for pi, par in enumerate(pars)]\n" 
 484          if verbose: 
 485              code += "  print sum(prods)\n" 
 486          code += "  return %s(sum(prods), %f)\n"%(op,this_tol) 
 487          exec code 
 488          constraint_funcs.append(locals()['D'+f.name]) 
 489   
 490      for func in constraint_funcs: 
 491          problem.addConstraint(FunctionConstraint(func), pars) 
 492   
 493      print "Use problem.getSolution() to find a solution" 
 494      return problem, D

 495   
 496   


497 -def norm_D_sum(D_sum):


498      """Normalize a D_sum by the elements for each feature by 
 499      smallest absolute size (that element becomes 1 in norm). 
 500      For unweighted feature sensitivities, or else it 
 501      unweights weighted ones.""" 
 502      D_n = {} 
 503      for feat, pD in D_sum.items(): 
 504          pD_min_abs = min(abs(array(pD.values()))) 
 505          D_n[feat] = {} 
 506          for pname, pD_val in pD.items(): 
 507              D_n[feat][pname] = pD_val/pD_min_abs 
 508      return D_n

 509   


510 -def filter_iface(psens, iface):


511      ws = {} 
 512      for parname, wdict in psens.items(): 
 513          ws[parname] = {iface: wdict[iface]} 
 514      return ws

 515   


516 -def organize_feature_sens(feat_sens, discrete_feats=None):


517      # Model interface is currently ignored -- assumes no clashing feature names 
 518      # between related MIs 
 519      if discrete_feats is None: 
 520          discrete_feats = [] 
 521      pars = feat_sens.keys() 
 522      pars.sort() 
 523   
 524      D_sum = {} 
 525      D_vec = {} 
 526   
 527      max_val = 0 
 528      for par in pars: 
 529          for mi, fdict in feat_sens[par].iteritems(): 
 530              for f, ra in fdict.iteritems(): 
 531                  val = sum(ra) 
 532                  if norm(val) > max_val: 
 533                      max_val = val 
 534                  if f not in D_sum and not(f in discrete_feats): 
 535                      D_sum[f] = {} 
 536                      D_vec[f] = {} 
 537                  if f in discrete_feats: 
 538                      continue 
 539                  else: 
 540                      D_sum[f][par] = val 
 541                      D_vec[f][par] = ra 
 542      return D_sum, D_vec

 543   
 544   


545 -def make_opt(pnames, resfnclass, model, context, parscales=None, 
 546               parseps=None, parstep=None, parlinesearch=None, 
 547               stopcriterion=None, grad_ratio_tol=10, 
 548               use_filter=False, verbose_level=2):


549      """Create a ParamEst manager object and an instance of an optimizer from the 
 550      Toolbox.optimize sub-package, returned as a pair. 
 551   
 552      Inputs: 
 553   
 554      pnames:     list of free parameters in the model 
 555      resfnclass: residual function class (e.g. residual_fn_context_1D exported 
 556                  from this module) 
 557      model:      the model to optimize, of type Model (not a Generator) 
 558      context:    the context object that defines the objective function 
 559                  criteria via "model interfaces" and their features, etc. 
 560      parscales:  for models that do not have parameters varying over similar 
 561                  scales, this dictionary defines what "O(1)" change in dynamics 
 562                  refers to for each parameter. E.g. a parameter that must change by 
 563                  several thousand in order to make an O(1) change in model output 
 564                  can have its scale set to 1000. This will also be the maximum 
 565                  step size in that direction for the Scaled Line Search method, if used. 
 566                  Defaults to 10*parseps for each parameter. 
 567      parseps:    dictionary to indicate what change in parameter value to use for 
 568                  forward finite differencing, for reasons similar to those given in 
 569                  description of the parscales argument. Default is 1e-7 for each parameter. 
 570      parstep:    choice of optimization algorithm stepper, defaults to 
 571                  conjugate gradient step.CWConjugateGradientStep. 
 572      parlinesearch:  choice of line search method, defaults to scaled 
 573                      line search method line_search.ScaledLineSearch. 
 574      stopcriterion:  choice of stop criteria for the optimization iterations. Defaults to 
 575                      ftol=1e-7, gtol=1e-7, iterations_max=200. 
 576      grad_ratio_tol: For residual functions with poor smoothness in some directions, 
 577                      this parameter (default = 10) prevents those directions being used 
 578                      for gradient information if the ratio of residual values found during 
 579                      finite differencing is greater in magnitude than this tolerance value. 
 580                      (Experimental option only -- set very large, e.g. 1e6 to switch off). 
 581      use_filter:     activate use of filtering out largest directions of gradients that may 
 582                      be unreliable. Default is False. (Experimental option only). 
 583      verbose_level:  Default to 2 (high verbosity). 
 584      """ 
 585      parnames = copy(pnames) 
 586      parnames.sort() 
 587      if parscales is None: 
 588          freepars = parnames 
 589      else: 
 590          freepars = filteredDict(parscales, parnames) 
 591      if parseps is None: 
 592          parseps = {}.fromkeys(parnames, 1e-7) 
 593      if parscales is None: 
 594          parscales = parseps.copy() 
 595          for k, v in parscales.iteritems(): 
 596              parscales[k] = 10*v 
 597      pest = ParamEst(freeParams=freepars, 
 598                   testModel=model, 
 599                   context=context, 
 600                   residual_fn=resfnclass(eps=[parseps[p] for p in parnames], 
 601                                          grad_ratio_tol=grad_ratio_tol), 
 602                   verbose_level=verbose_level 
 603                  ) 
 604      if parstep is None: 
 605          parstep = step.CWConjugateGradientStep() 
 606      if parlinesearch is None: 
 607          parlinesearch = line_search.ScaledLineSearch(max_step=[parscales[p] for \ 
 608                                                  p in parnames], filter=use_filter) 
 609      if stopcriterion is None: 
 610          stopcriterion = criterion.criterion(ftol=1e-7, gtol=1e-7, 
 611                                iterations_max=200) 
 612      return pest, optimizer.StandardOptimizer(function=pest.fn, 
 613                      step=parstep, 
 614                      line_search=parlinesearch, 
 615                      criterion=stopcriterion, 
 616                      x0=pest.pars_dict_to_array(pest.testModel.pars))

 617   
 618   


619 -def restrict_opt(pest, feat_list, opt, pars=None):


620      """Restrict parameter estimation to certain features and parameters. 
 621   
 622      If pars is None (default) then all free parameters of pest are used. 
 623      """ 
 624      if pars is None: 
 625          pars = pest.freeParNames 
 626      if parseps is None: 
 627          parseps = {}.fromkeys(pest.freeParNames, 1e-7) 
 628      if parstep is None: 
 629          parstep = step.CWConjugateGradientStep() 
 630      if parlinesearch is None: 
 631          parlinesearch = line_search.ScaledLineSearch(max_step = \ 
 632                                  [pest.parScales[p] for p in pars]) 
 633      if stopcriterion is None: 
 634          stopcriterion = criterion.criterion(ftol=1e-7, gtol=1e-7, 
 635                                iterations_max=100) 
 636      new_pest = ParamEst(context=pest.context, 
 637                          freeParams=filteredDict(pest.parScales, pars), 
 638                          testModel=pest.testModel, 
 639                          verbose_level=pest.verbose_level) 
 640      new_fn = pest.fn.__class__(eps=[parseps[p] for p in pars], 
 641                                 pest=new_pest) 
 642      new_pest.setFn(new_fn) 
 643  #    full_feat_list = pest.context.res_feature_list 
 644  #    wdict = {} 
 645  #    for mi, feat in full_feat_list: 
 646  #        if (mi, feat) not in feat_list: 
 647  #            try: 
 648  #                wdict[mi][feat] = 0 
 649  #            except KeyError: 
 650  #                wdict[mi] = {feat: 0} 
 651      # leave weights for selected features at their previous values from 
 652      # pest.context 
 653  #    new_pest.context.set_weights(wdict) 
 654      new_pest.fn.pest = new_pest   # otherwise logging goes to wrong place 
 655      new_opt = optimizer.StandardOptimizer(function=new_pest.fn, step=parstep, 
 656                              line_search=parlinesearch, 
 657                              criterion=stopcriterion, 
 658                              x0=new_pest.pars_dict_to_array(pest.testModel.pars)) 
 659      return new_pest, new_opt

 660   
 661   
 662   


663 -class L2_feature_1D(qt_feature_leaf):


664      """Use with scalar optimizers such as BoundMin""" 


665 -    def _local_init(self):


666          self.metric = metric_L2_1D() 
 667          if hasattr(self.pars, 'num_samples'): 
 668              self.metric_len = self.pars.num_samples 
 669          else: 
 670              self.metric_len = len(self.pars.t_samples)

 671   


672 -    def postprocess_ref_traj(self):


673          if hasattr(self.pars, 'num_samples'): 
 674              tvals = linspace(self.pars.trange[0], self.pars.trange[1], 
 675                           self.metric_len) 
 676          else: 
 677              tvals = self.pars.t_samples 
 678          self.pars.tvals = tvals 
 679          self.pars.ref_samples = self.ref_traj(tvals, coords=[self.pars.coord])

 680   


681 -    def evaluate(self, target):


682          return self.metric(self.pars.ref_samples, 
 683                             target.test_traj(self.pars.tvals, 
 684                                              self.pars.coord)) < self.pars.tol

 685   


686 -class L2_feature(qt_feature_leaf):


687 -    def _local_init(self):


688          self.metric = metric_L2() 
 689          if hasattr(self.pars, 'num_samples'): 
 690              self.metric_len = self.pars.num_samples 
 691          else: 
 692              self.metric_len = len(self.pars.t_samples)

 693   


694 -    def postprocess_ref_traj(self):


695          if hasattr(self.pars, 'num_samples'): 
 696              tvals = linspace(self.pars.trange[0], self.pars.trange[1], 
 697                           self.metric_len) 
 698          else: 
 699              tvals = self.pars.t_samples 
 700          self.pars.tvals = tvals 
 701          self.pars.ref_samples = self.ref_traj(tvals, coords=[self.pars.coord])

 702   


703 -    def evaluate(self, target):


704          return self.metric(self.pars.ref_samples, 
 705                             target.test_traj(self.pars.tvals, 
 706                                              self.pars.coord)) < self.pars.tol

 707   
 708   


709 -class ParamEst(Utility):


710      """General-purpose parameter estimation class. 
 711      freeParams keyword initialization argument may be a list of 
 712      names or a dictionary of scales for determining appropriate 
 713      step sizes for O(1) changes in the residual function. 
 714   
 715      In its absence, the scales will default to 1. 
 716      """ 
 717   


718 -    def __init__(self, **kw):


719          self.needKeys = ['freeParams', 'testModel', 'context'] 
 720          self.optionalKeys = ['verbose_level', 'usePsyco', 
 721                               'residual_fn', 'extra_pars'] 
 722          try: 
 723              self.context = kw['context'] 
 724              if isinstance(kw['freeParams'], list): 
 725                  self.freeParNames = kw['freeParams'] 
 726                  self.numFreePars = len(self.freeParNames) 
 727                  self.parScales = dict.fromkeys(self.freeParNames, 1) 
 728              else: 
 729                  self.parScales = kw['freeParams'] 
 730                  self.freeParNames = self.parScales.keys() 
 731                  self.numFreePars = len(self.freeParNames) 
 732              self.freeParNames.sort() 
 733              self.testModel = kw['testModel'] 
 734              assert isinstance(self.testModel, Model), \ 
 735                     "testModel argument must be a Model instance" 
 736              self._algParamsSet = False 
 737          except KeyError: 
 738              raise PyDSTool_KeyError('Incorrect argument keys passed') 
 739          self.foundKeys = len(self.needKeys)   # lazy way to achieve this! 
 740          if 'usePsyco' in kw: 
 741              if HAVE_PSYCO and kw['usePsyco']: 
 742                  self.usePsyco = True 
 743              else: 
 744                  self.usePsyco = False 
 745              self.foundKeys += 1 
 746          else: 
 747              self.usePsyco = False 
 748          if 'residual_fn' in kw: 
 749              self.setFn(kw['residual_fn']) 
 750              self.foundKeys += 1 
 751          else: 
 752              try: 
 753                  res_fn = res_fn_lookup[self.__class__] 
 754              except KeyError: 
 755                  raise ValueError("Must explicitly set residual function for this class") 
 756              else: 
 757                  self.setFn(res_fn(pest=self)) 
 758          if 'extra_pars' in kw: 
 759              self._extra_pars = kw['extra_pars'] 
 760              self.foundKeys += 1 
 761          else: 
 762              self._extra_pars = {} 
 763          self.parsOrig = {} 
 764          # in case explicit jacobian of residual fn is not present 
 765          self._residual_fn_jac = None 
 766          if 'verbose_level' in kw: 
 767              self.verbose_level = kw['verbose_level'] 
 768              self.foundKeys += 1 
 769          else: 
 770              self.verbose_level = 0 
 771          # Set up model arguments (parameter value will be set before needed) 
 772          self.modelArgs = {} 
 773          self.resetParArgs() 
 774          # used for Ridders' method output statistics if selected for 
 775          # calculating gradient using gradient or Hessian methods 
 776          self._grad_info = {} 
 777          if self.foundKeys < len(kw): 
 778              raise PyDSTool_KeyError('Incorrect argument keys passed') 
 779          self.reset_log()

 780   
 781   


782 -    def resetParArgs(self):


783          self.parTypeStr = [] 
 784          for i in xrange(self.numFreePars): 
 785              if self.freeParNames[i] in self.testModel.obsvars: 
 786                  # for varying initial conditions 
 787                  self.parTypeStr.append('ics') 
 788                  if 'ics' not in self.modelArgs: 
 789                      self.modelArgs['ics'] = {} 
 790              elif self.freeParNames[i] in self.testModel.pars: 
 791                  # for varying regular pars 
 792                  self.parTypeStr.append('pars') 
 793                  if 'pars' not in self.modelArgs: 
 794                      self.modelArgs['pars'] = {} 
 795              else: 
 796                  raise ValueError("free parameter '"+self.freeParNames[i]+"'"\ 
 797                                     " not found in test model") 
 798              # initialize model argument to None 
 799              self.modelArgs[self.parTypeStr[i]][self.freeParNames[i]] = None

 800   
 801   


802 -    def setAlgParams(self, *args):


803          """Set algorithmic parameters.""" 
 804          raise NotImplementedError("This is only an abstract function " 
 805                                    "definition")

 806   


807 -    def setFn(self, fn):


808          self.fn = fn 
 809          # reciprocal reference 
 810          self.fn.pest = self 
 811          if self.usePsyco: 
 812              psyco.bind(self.fn)

 813   
 814   


815 -    def evaluate(self, extra_record_info=None):


816          """Evaluate residual vector, record result, and display step 
 817          information (if verbose). 
 818          """ 
 819          res, raw_res = self.context.residual(self.testModel, include_raw=True) 
 820          log_entry = args(pars=filteredDict(self.testModel.query('pars'), 
 821                                                   self.freeParNames), 
 822                               ics=filteredDict(self.testModel.query('ics'), 
 823                                                self.freeParNames), 
 824                               weights=self.context.weights, 
 825                               residual_vec=res, 
 826                               raw_residual_vec=raw_res, 
 827                               residual_norm=norm(res), 
 828                               trajectories=[copy(ref_mi.get_test_traj()) for \ 
 829                                ref_mi in self.context.ref_interface_instances]) 
 830          self.log.append(log_entry) 
 831          key = {} 
 832          key.update(log_entry.pars) 
 833          key.update(log_entry.ics) 
 834          self._keylogged[tuple(sortedDictValues(key))] = log_entry 
 835          if extra_record_info is not None: 
 836              self.log[-1].update({'extra_info': args(**extra_record_info)}) 
 837          if norm(res) < self.log[self._lowest_res_log_ix].residual_norm: 
 838              # this is now the lowest recorded, so make note of this index into self.log 
 839              self._lowest_res_log_ix = len(self.log)-1 
 840          if self.verbose_level > 0: 
 841              self.show_log_record(self.iteration) 
 842          self.iteration += 1 
 843          return res

 844   
 845   


846 -    def reset_log(self):


847          self.iteration = 0 
 848          self.log = [] 
 849          self._lowest_res_log_ix = 0 
 850          # key logged is used for faster cache lookup using pars + ics 
 851          self._keylogged = {}

 852   


853 -    def key_logged_residual(self, pars_ics, weights):


854          """pars_ics must be a sequence type""" 
 855          try: 
 856              log_entry = self._keylogged[tuple(pars_ics)] 
 857          except KeyError: 
 858              raise KeyError("Pars and ICs not found in log record") 
 859          if all(log_entry.weights == weights): 
 860              return log_entry.residual_vec 
 861          else: 
 862              return process_raw_residual(log_entry.raw_residual_vec, weights)

 863   
 864   


865 -    def find_logs(self, res_val=None, condition='<'):


866          """Find log entries matching given condition on their residual norm 
 867          values. Returns a list of log indices. 
 868   
 869          if res_val is not given, the residual norm of the first entry in the 
 870          current log is used. 
 871   
 872          Use '<' and '>' for the condition argument (default is <). 
 873          """ 
 874          if res_val is None: 
 875              res_val = self.log[0].residual_norm 
 876          res_data = array([e.residual_norm for e in self.log]) 
 877          sort_ixs = argsort(res_data) 
 878          if condition == '<': 
 879              ix = argmin(res_data[sort_ixs] < res_val) 
 880              return list(sort_ixs[:ix]) 
 881          elif condition == '>': 
 882              ix = argmax(res_data[sort_ixs] > res_val) 
 883              return list(sort_ixs[ix:])

 884   


885 -    def show_log_record(self, i, full=False):


886          """Use full option to show residuals mapped to their feature names, 
 887          including information about weights.""" 
 888          try: 
 889              entry = self.log[i] 
 890          except IndexError: 
 891              raise ValueError("No such call %i recorded"%i) 
 892          print "\n  **** Call %i"%i, "Residual norm: %f"%entry.residual_norm 
 893          if entry.ics != {}: 
 894              print "Ics:", entry.ics 
 895          if entry.pars != {}: 
 896              print "Pars:", entry.pars 
 897          if full: 
 898              print "Res:\n" 
 899              self.context.show_res_info(entry.residual_vec) 
 900          else: 
 901              print "Res:", entry.residual_vec

 902   


903 -    def pars_to_ixs(self):


904          all_pars = sortedDictKeys(self.testModel.pars) 
 905          inv_ixs = [all_pars.index(p) for p in self.freeParNames] 
 906          inv_ixs.sort() 
 907          return inv_ixs

 908   


909 -    def pars_array_to_dict(self, parray):


910          return dict(zip(self.freeParNames, parray))

 911   


912 -    def pars_dict_to_array(self, pdict):


913          return array(sortedDictValues(filteredDict(pdict, self.freeParNames)))

 914   


915 -    def par_sensitivity(self, pdict=None, non_diff_feats=None, extra_info=False):


916          """Parameter sensitivity of the context's features at the free parameter 
 917          values given as a dictionary or args. If none provided, the current 
 918          test model parameter values will be used. A dictionary mapping parameter names to 
 919            {interface_instance: {feat1: sensitivity_array, ..., featn: sensitivity_array}} 
 920          is returned. 
 921   
 922          Specify any non-differentiable features in the non_diff_feats list 
 923          as pairs (interface instance, feature instance). 
 924   
 925          Sensitivity entry > 0 means that increasing the parameter will 
 926          increase the absolute value of that residual, i.e. worsen the "fit". 
 927   
 928          extra_info optional argument makes this method return both the feature sensitivity 
 929          dictionary and a dictionary containing additional information to reconstruct 
 930          the gradient of the residual norm, to save re-calculation of it at this point. 
 931          This gradient will also respect the non_diff_feats argument, if provided. 
 932          """ 
 933          old_weights = self.context.weights 
 934          self.context.reset_weights() 
 935          wdict={} 
 936          if non_diff_feats is not None: 
 937              for mi, f in non_diff_feats: 
 938                  if mi in wdict: 
 939                      wdict[mi][f] = 0 
 940                  else: 
 941                      wdict[mi] = {f:0} 
 942          self.context.set_weights(wdict) 
 943          if pdict is None: 
 944              pdict = filteredDict(self.testModel.pars, self.freeParNames) 
 945          p = self.pars_dict_to_array(pdict) 
 946          f = self.fn.residual 
 947          res = f(p) 
 948          if extra_info: 
 949              info_dict = {'res': res} 
 950              res_dict = {} 
 951              grad_dict = {} 
 952          feat_sens = {} 
 953          for pi, pn in enumerate(self.freeParNames): 
 954              p_copy = p.copy() 
 955              try: 
 956                  h = self.fn.eps[pi] 
 957              except TypeError: 
 958                  # scalar 
 959                  h = self.fn.eps 
 960              p_copy[pi] += h 
 961              res_eps = f(p_copy) 
 962              # multiple and check inclusive inequality in case sign_res has 
 963              # components at exactly 0 
 964              assert alltrue(res * res_eps >= 0), "step for %s too large"%pn 
 965              D_res = (res_eps-res)/h 
 966              if extra_info: 
 967                  res_dict[pn] = (res_eps, h) 
 968                  grad_dict[pn] = (norm(old_weights*res_eps)-norm(old_weights*res))/h 
 969              feat_sens[pn] = self.context._map_to_features(D_res) 
 970          self.context.reset_weights(old_weights) 
 971          if extra_info: 
 972              info_dict['res_dict'] = res_dict 
 973              info_dict['weights'] = old_weights 
 974              info_dict['grad'] = grad_dict 
 975              return feat_sens, info_dict 
 976          else: 
 977              return feat_sens

 978   


979 -    def weighted_par_sensitivity(self, feat_sens):


980          """Return parameter sensitivities weighted according to current feature 
 981          weights, based on a previous output from par_sensitivity method. 
 982          """ 
 983          ws = self.context.feat_weights 
 984          wfeat_sens = {} 
 985          for pn, sensdict in feat_sens.iteritems(): 
 986              pd = wfeat_sens[pn] = {} 
 987              for mi, fdict in sensdict.iteritems(): 
 988                  md = pd[mi] = {} 
 989                  for f, sens in fdict.iteritems(): 
 990                      md[f] = sens*ws[(mi,f)] 
 991          return wfeat_sens

 992   


993 -    def run(self):


994          """Run parameter estimation. Returns a dictionary: 
 995   
 996              'success' -> boolean 
 997              'pars_sol' -> fitted values of pars 
 998              'pars_orig' -> original values of optimized pars 
 999              'sys_sol' -> trajectory of best fit Model trajectory 
1000              'alg_results' -> all other algorithm information (list) 
1001          """ 
1002          raise NotImplementedError("This is only an abstract method definition")

1003   


1004 -    def iterate(self):


1005          raise NotImplementedError("This is only an abstract method definition")

1006   
1007   


1008 -class LMpest(ParamEst):


1009      """Unconstrained least-squares parameter and initial condition optimizer 
1010      for n-dimensional DS trajectories. Fits N-dimensional parameter spaces. 
1011   
1012      Uses MINPACK Levenberg-Marquardt algorithm wrapper from SciPy.minimize. 
1013      """ 
1014   


1015 -    def setAlgParams(self, changed_parDict=None):


1016          # defaults 
1017          parDict = { 
1018                   'residuals' : None, 
1019                   'p_start'   : None, 
1020                   'args'      : None, 
1021                   'Dfun'      : None, 
1022                   'full_output' : 1, 
1023                   'col_deriv'   : 0, 
1024                   'ftol'        : 5e-5, 
1025                   'xtol'        : 5e-5, 
1026                   'gtol'        : 0.0, 
1027                   'maxfev'      : 100, 
1028                   'epsfcn'      : 0.0, 
1029                   'factor'      : 100, 
1030                   'diag'        : None 
1031                   } 
1032   
1033          if changed_parDict is None: 
1034              changed_parDict = {} 
1035          parDict.update(copy(changed_parDict)) 
1036          assert len(parDict) == 13, "Incorrect param dictionary keys used" 
1037   
1038          self._residuals   = parDict['residuals'] 
1039          self._p_start     = parDict['p_start'] 
1040          self._args        = parDict['args'] 
1041          self._Dfun        = parDict['Dfun'] 
1042          self._full_output = parDict['full_output'] 
1043          self._col_deriv   = parDict['col_deriv'] 
1044          self._ftol        = parDict['ftol'] 
1045          self._xtol        = parDict['xtol'] 
1046          self._gtol        = parDict['gtol'] 
1047          self._maxfev      = parDict['maxfev'] 
1048          self._epsfcn      = parDict['epsfcn'] 
1049          self._factor      = parDict['factor'] 
1050          self._diag        = parDict['diag'] 
1051          # flag for run() to start 
1052          self._algParamsSet = True

1053   
1054   


1055 -    def run(self, parDict=None, extra_pars=None, verbose=False):


1056          """Begin parameter estimation run. 
1057   
1058          parDict can include arbitrary additional runtime arguments to 
1059          the residual function. 
1060   
1061          If tmesh is not supplied an attempt will be made to create one 
1062          from the goal trajectory's independent domain limits, if the 
1063          trajectory has been provided. Default mesh resolution is 20 points. 
1064          """ 
1065          if parDict is None: 
1066              parDict_new = {} 
1067          else: 
1068              parDict_new = copy(parDict) 
1069          self._extra_pars = extra_pars 
1070          parsOrig = [] 
1071          self.numFreePars = len(self.freeParNames) 
1072          self.resetParArgs() 
1073          for i in xrange(self.numFreePars): 
1074              val = self.testModel.query(self.parTypeStr[i])\ 
1075                              [self.freeParNames[i]] 
1076              self.parsOrig[self.freeParNames[i]] = val 
1077              parsOrig.append(val) 
1078          parsOrig = array(parsOrig) 
1079          parDict_new['p_start'] = copy(parsOrig) 
1080          if 'residuals' not in parDict_new: 
1081              parDict_new['residuals'] = self.fn.residual 
1082          if 'Dfun' not in parDict_new: 
1083              # may be None 
1084              if not isinstance(self.fn, helpers.FiniteDifferencesFunction): 
1085                  parDict_new['Dfun'] = self.fn.jacobian 
1086   
1087          # Setting default minimizer pars 
1088  ##        if not self._algParamsSet: 
1089          self.setAlgParams(parDict_new) 
1090   
1091          self.reset_log() 
1092          # perform least-squares fitting 
1093          if not verbose: 
1094              rout.start() 
1095              rerr.start() 
1096          try: 
1097              results = minpack.leastsq(self._residuals, 
1098                                 self._p_start, 
1099                                 args   = self._args, 
1100                                 Dfun   = self._Dfun, 
1101                                 full_output = self._full_output, 
1102                                 col_deriv   = self._col_deriv, 
1103                                 ftol   = self._ftol, 
1104                                 xtol   = self._xtol, 
1105                                 gtol   = self._gtol, 
1106                                 maxfev = self._maxfev, 
1107                                 epsfcn = self._epsfcn, 
1108                                 factor = self._factor, 
1109                                 diag   = self._diag) 
1110          except: 
1111              if not verbose: 
1112                  out = rout.stop() 
1113                  err = rerr.stop() 
1114              print "Calculating residual failed for pars:", \ 
1115                    parsOrig 
1116              raise 
1117          if not verbose: 
1118              out = rout.stop() 
1119              err = rerr.stop() 
1120   
1121          # build return information 
1122          success = results[4] == 1 
1123          if isinstance(results[0], float): 
1124              res_par_list = [results[0]] 
1125              orig_par_list = [parsOrig[0]] 
1126          else: 
1127              res_par_list = results[0].tolist() 
1128              orig_par_list = parsOrig.tolist() 
1129          alg_results = results[2] 
1130          alg_results['message'] = results[3] 
1131          self.pestResult = {'success': success, 
1132                        'cov': results[1], 
1133                        'pars_sol': dict(zip(self.freeParNames, 
1134                                             res_par_list)), 
1135                        'pars_orig': dict(zip(self.freeParNames, 
1136                                              orig_par_list)), 
1137                        'alg_results': alg_results, 
1138                        'sys_sol': self.testModel 
1139                        } 
1140   
1141          if verbose: 
1142              # This is a very output-sensitive hack for finding instances where 
1143              # the algorithm stopped because it reached tolerances, not 
1144              # because it converged. 
1145              if success or results[3].find('at most') != -1: 
1146                  if success: 
1147                      print 'Solution of ', self.freeParNames, ' = ', results[0] 
1148                  else: 
1149  ##                    parvals = [self.testModel.pars[p] for p in \ 
1150  ##                               self.freeParNames] 
1151                      print 'Closest values of ', self.freeParNames, ' = ', \ 
1152                            results[0] 
1153  ##                          parvals 
1154                  print 'Original values = ', parsOrig 
1155                  print 'Number of fn evals = ', results[2]["nfev"], \ 
1156                               '(# iterations)' 
1157                  if not success: 
1158                      print 'Solution not found: '+results[3] 
1159              else: 
1160                  print 'Solution not found: '+results[3] 
1161          return copy(self.pestResult)

1162   
1163   


1164 -    def _make_res_float(self, pars):


1165          """Returns a function that converts residual vector to its norm 
1166          (a single floating point total residual). 
1167   
1168          (Helper method for gradient and Hessian) 
1169          """ 
1170          def _residual_float(x): 
1171              return Point({'r': \ 
1172                        self.fn(array([x[n] for n in pars], 'd'))})

1173          return _residual_float

1174   
1175   


1176 -    def gradient_total_residual(self, x, eps=None, pars=None, use_ridder=False):


1177          """Compute gradient of total residual (norm of the residual function) 
1178          at x as a function of parameter names specified (defaults to all 
1179          free parameters). 
1180          """ 
1181          if pars is None: 
1182              pars = self.freeParNames 
1183          if eps is None: 
1184              eps = self.fn.eps 
1185          if use_ridder: 
1186              # Ridders' method (more accurate, slower) 
1187              return common.diff(self._make_res_float(pars), 
1188                          Point(filteredDict(x, pars)), 
1189                          vars=pars, eps=eps, output=self._grad_info) 
1190          else: 
1191              # regular finite differences 
1192              return common.diff2(self._make_res_float(pars), 
1193                          Point(filteredDict(x, pars)), 
1194                          vars=pars, eps=eps)

1195   
1196   


1197 -    def Hessian_total_residual(self, x, eps_inner=None, eps_outer=None, 
1198                  pars=None, use_ridder_inner=False, use_ridder_outer=False):


1199          """Compute Hessian of total residual (norm of the residual function) 
1200          at x as a function of parameter names specified (defaults to all 
1201          free parameters), USING FINITE DIFFERENCES. 
1202   
1203          Option to use different eps scalings for the inner gradient 
1204          calculations versus the outer gradient of those values. 
1205   
1206          It might be more accurate to calculate the Hessian using a QR 
1207          decomposition of the Jacobian. 
1208          """ 
1209          if pars is None: 
1210              pars = self.freeParNames 
1211          res_fn = self._make_res_float(pars) 
1212          if use_ridder_inner: 
1213              diff_inner = common.diff 
1214          else: 
1215              diff_inner = common.diff2 
1216   
1217          def Dfun(x): 
1218              diffx=array(diff_inner(res_fn, Point(filteredDict(x, pars)), 
1219                                   vars=pars, eps=eps_inner)) 
1220              diffx.shape=(len(pars),) 
1221              return Point(coordarray=diffx, coordnames=pars)

1222   
1223          if use_ridder_outer: 
1224              # Ridders' method (more accurate, slower) 
1225              return common.diff(Dfun, 
1226                          Point(filteredDict(x, pars)), 
1227                          vars=pars, eps=eps_outer, output=self._grad_info) 
1228          else: 
1229              # regular finite differences 
1230              return common.diff2(Dfun, 
1231                          Point(filteredDict(x, pars)), 
1232                          vars=pars, eps=eps_outer) 
1233   
1234   
1235   
1236   


1237 -class BoundMin(ParamEst):


1238      """Bounded minimization parameter and initial condition optimizer 
1239      for one-dimensional DS trajectories. Fits 1 parameter only. 
1240   
1241      Uses SciPy.optimize fminbound algorithm. 
1242      """ 
1243   


1244 -    def __init__(self, **kw):


1245          assert len(kw['freeParams']) == 1, ("Only one free parameter can " 
1246                                              "be specified for this class") 
1247          ParamEst.__init__(self, **kw) 
1248          if self.freeParNames[0] in self.testModel.obsvars: 
1249              # for varying initial conditions 
1250              self.parTypeStr = 'ics' 
1251          elif self.freeParNames[0] in self.testModel.pars: 
1252              # for varying regular pars 
1253              self.parTypeStr = 'pars' 
1254          else: 
1255              raise ValueError('free parameter name not found in test model') 
1256          # Set up model arguments (parameter value will be set before needed) 
1257          self.modelArgs = {self.parTypeStr: {self.freeParNames[0]: None}}

1258   
1259   


1260 -    def run(self, parConstraints, xtol=5e-5, maxiter=500, 
1261              extra_args=(), verbose=False):


1262          val = self.testModel.query(self.parTypeStr)[self.freeParNames[0]] 
1263          self.parsOrig = {self.freeParNames[0]: val} 
1264          parsOrig = val 
1265   
1266          self.reset_log() 
1267          full_output = 1 
1268          if not verbose: 
1269              rout.start() 
1270              rerr.start() 
1271          try: 
1272              results = optimize.fminbound(self.fn.residual, parConstraints[0], 
1273                          parConstraints[1], extra_args, xtol, maxiter, 
1274                                       full_output, 
1275                                       int(verbose)) 
1276          except: 
1277              if not verbose: 
1278                  out = rout.stop() 
1279                  err = rerr.stop() 
1280              raise 
1281          else: 
1282              if not verbose: 
1283                  out = rout.stop() 
1284                  err = rerr.stop() 
1285   
1286          # build return information 
1287          success = results[2] == 0 
1288          self.pestResult = {'success': success, 
1289                        'pars_sol': {self.freeParNames[0]: results[0]}, 
1290                        'pars_orig': {self.freeParNames[0]: parsOrig}, 
1291                        'alg_results': results[3], 
1292                        'sys_sol': self.testModel 
1293                        } 
1294   
1295          if verbose: 
1296              if success: 
1297                  print 'Solution of ', self.freeParNames[0], ' = ', results[0] 
1298                  print 'Original value = ', parsOrig 
1299                  print 'Number of fn evals = ', results[3], "(# iterations)" 
1300                  print 'Error tolerance = ', xtol 
1301              else: 
1302                  print 'No convergence of BoundMin' 
1303                  print results 
1304          return copy(self.pestResult)

1305   
1306   
1307   
1308  res_fn_lookup = {LMpest: residual_fn_context, 
1309                   BoundMin: residual_fn_context_1D} 
1310   
1311  # ---------------------------------------------------------------------------- 
1312   
1313  ## DEPRECATED FUNCTIONS 
1314  ## Utility functions for estimation using objective functions measuring 
1315  # extrema locations. These functions assume the presence of events to 
1316  # detect extrema during generation of test trajectories. 
1317   


1318 -def get_slope_info(x, lookahead=1, prec=1e-3, default=1):


1319      """DEPRECATED. Use features - they work more efficiently and robustly. 
1320      e.g. see Toolbox/neuro_data.py 
1321   
1322      Helper function for qualitative fitting. 
1323   
1324      Local slope information about data array x. Values of 1 in the 
1325      return array indicate increasing slopes over a local extent given 
1326      by the the lookahead argument, whereas 0 indicates non-increasing 
1327      slopes. 
1328   
1329      The default value specifies the value taken by the returned 
1330      array in the indices from len(x)-lookahead to len(x). 
1331      """ 
1332      if default==1: 
1333          s = ones(shape(x), 'float') 
1334      elif default==0: 
1335          s = zeros(shape(x), 'float') 
1336      else: 
1337          raise ValueError("Use default = 0 or 1 only") 
1338      for i in xrange(len(x)-lookahead): 
1339          s[i,:] = [max(prec,val) for val in \ 
1340                    ravel((x[i+lookahead,:]-x[i,:]).toarray())] 
1341      return s

1342   
1343   


1344 -def get_extrema(x, t, tmin, tmax, coords, per, pertol_frac, 
1345                  lookahead, lookahead_tol, fit_fn_class=None, 
1346                  verbose=False):


1347      """DEPRECATED. Use features - they work more efficiently and robustly. 
1348      e.g. see Toolbox/neuro_data.py 
1349   
1350      Helper function for qualitative fitting. 
1351   
1352      per is an estimate of the period, per <= tmax. 
1353      pertol_frac is fraction of period used as tolerance for finding extrema. 
1354      fit_fn_class switches on interpolation of extremum by the fitting of a 
1355            local function (uses least squares criterion) - specify a sub-class 
1356            of fit_function (default None). 
1357      """ 
1358      # want slope lookahead to be smaller than noise-avoidance lookahead 
1359      slope_lookahead = max([2, lookahead/2]) 
1360      slopes = Pointset(coordarray=transpose(get_slope_info(x[coords], 
1361                                       slope_lookahead, prec=0, default=0))>0, 
1362                        coordnames=coords, 
1363                        indepvararray=t) 
1364      maxs_t = {}.fromkeys(coords) 
1365      mins_t = {}.fromkeys(coords) 
1366      maxs_v = {}.fromkeys(coords) 
1367      mins_v = {}.fromkeys(coords) 
1368      last_t = {}.fromkeys(coords) 
1369      last_type = {}.fromkeys(coords) 
1370      detect_on = [] 
1371      for c in coords: 
1372          maxs_t[c] = [] 
1373          mins_t[c] = [] 
1374          maxs_v[c] = [] 
1375          mins_v[c] = [] 
1376          last_t[c] = [-1,-1] 
1377          last_type[c] = -1 
1378          detect_on.append([True,True])   # False when outside of pertol_frac tolerance for each max and min 
1379      assert pertol_frac < 1 and pertol_frac > 0 
1380      assert per > 0 and per <= tmax 
1381      assert tmin > t[0] and tmax < t[-1] 
1382      detect_tol = pertol_frac*per 
1383      halfper=per/2. 
1384      max_ix = len(t)-1 
1385      ms={0:'min', 1:'max'} 
1386      res = x.find(tmin) 
1387      if isinstance(res, tuple): 
1388          tix_lo = res[0] 
1389      else: 
1390          tix_lo = res 
1391      res = x.find(tmax) 
1392      if isinstance(res, tuple): 
1393          tix_hi = res[0] 
1394      else: 
1395          tix_hi = res 
1396      if fit_fn_class is None: 
1397          do_fit = False 
1398      else: 
1399          do_fit = True 
1400          fit_fn = fit_fn_class() 
1401      # provide some initial history for slope detection info at t = tmin - dt 
1402      last_inc = slopes.coordarray[:,tix_lo-1] 
1403      for local_ix, tval in enumerate(t[tix_lo:tix_hi]): 
1404          ival = local_ix + tix_lo 
1405          ival_ml = max([0, ival - lookahead]) 
1406          ival_pl = min([max_ix, ival + lookahead]) 
1407          if verbose: 
1408              print "*********** t =", tval, " , ival =", ival 
1409          v = slopes.coordarray[:,ival] 
1410          for ci, c in enumerate(coords): 
1411              for m in [0,1]: 
1412                  if detect_on[ci][m]: 
1413                      pass 
1414  ##                    if last_t[c][m]>0: 
1415  ##                        if tval-last_t[c][m]+per+detect_tol > detect_tol: 
1416  ##                            detect_on[ci][m] = False 
1417  ##                            print " - %s detect for %s now False:"%(ms,c), last_t[c][m], detect_tol 
1418                  else: 
1419                      if tval>(last_t[c][m]+per-detect_tol): 
1420                          if not detect_on[ci][1-m] and 1-m == last_type[ci]: 
1421                              if tval>(last_t[c][1-m]+halfper-detect_tol): 
1422                                  detect_on[ci][m] = True 
1423                                  if verbose: 
1424                                      print " + %s detect (>half per of %s) for %s now True:"%(ms[m],ms[1-m],c), last_t[c][1-m], last_t[c][1-m]+halfper-detect_tol 
1425                          # The next segment allows consecutive local extrema of the 
1426                          # same type without an intermediate of the other type. 
1427                          # This is generally not desirable! 
1428  ##                        else: 
1429  ##                        detect_on[ci][m] = True 
1430  ##                        if verbose: 
1431  ##                            print " + %s detect (>per) for %s now True:"%(ms[m],c), last_t[c][m], last_t[c][m]+per-detect_tol 
1432                      # The next segment allows consecutive local extrema of the 
1433                      # same type without an intermediate of the other type. 
1434                      # This is generally not desirable! 
1435  ##                    elif tval>(last_t[c][m]+halfper-detect_tol) and m==last_type[ci] and not detect_on[ci][1-m]: 
1436  ##                        detect_on[ci][1-m] = True 
1437  ##                        if verbose: 
1438  ##                            print " + %s detect (>half per) for %s now True:"%(ms[1-m],c), last_t[c][1-m], last_t[c][m]+halfper-detect_tol 
1439              do_anything = detect_on[ci][0] or detect_on[ci][1] 
1440              if verbose: 
1441                  print "Detecting for %s? (min=%i) (max=%i)"%(c, int(detect_on[ci][0]), int(detect_on[ci][1])) 
1442                  print "   v[ci] = %.4f, last_inc[ci] = %.4f"%(v[ci], last_inc[ci]) 
1443              if do_anything and v[ci] != last_inc[ci]: 
1444                  # extremum if changed sign 
1445                  if v[ci]>0: 
1446                      # - +  => min 
1447                      if detect_on[ci][0]: 
1448                          min_ival = argmin(x[c][ival:ival_pl])+ival 
1449                          if verbose: 
1450                              print "Possible min:" 
1451                              print x[c][max([0,min_ival-lookahead])], x[c][min_ival], x[c][min([max_ix,min_ival+lookahead])] 
1452                          if x[c][min([max_ix,min_ival+lookahead])] - x[c][min_ival] > lookahead_tol and \ 
1453                             x[c][max([0,min_ival-lookahead])] - x[c][min_ival] > lookahead_tol: 
1454                              if do_fit: 
1455                                  ixs_lo = max([0,min_ival-int(lookahead/4.)]) 
1456                                  ixs_hi = min([max_ix,min_ival+int(lookahead/4.)]) 
1457                                  res = fit_fn.fit(x['t'][ixs_lo:ixs_hi], 
1458                                                        x[c][ixs_lo:ixs_hi]) 
1459                                  xs_fit = res.ys_fit 
1460                                  p = res.pars_fit 
1461                                  min_tval, min_xval = res.results.peak 
1462                              else: 
1463                                  min_tval = x['t'][min_ival] 
1464                                  min_xval = x[c][min_ival] 
1465                              if verbose: 
1466                                  print "found min for %s at "%c, min_tval 
1467                              mins_t[c].append(min_tval) 
1468                              mins_v[c].append(min_xval) 
1469                              last_t[c][0]=min_tval 
1470                              detect_on[ci][0] = False 
1471                              detect_on[ci][1] = False 
1472                              last_type[ci]=0 
1473  ##                    else: 
1474  ##                        print "    ... ignoring b/c not detecting" 
1475                  else: 
1476                      # + - => max 
1477                      if detect_on[ci][1]: 
1478                          max_ival = argmax(x[c][ival:ival_pl])+ival 
1479                          if verbose: 
1480                              print "Possible max:" 
1481                              print x[c][max([0,max_ival-lookahead])], x[c][max_ival], x[c][min([max_ix,max_ival+lookahead])] 
1482                          if x[c][max_ival] - x[c][min([max_ix,max_ival+lookahead])] > lookahead_tol and \ 
1483                             x[c][max_ival] - x[c][max([0,max_ival-lookahead])] > lookahead_tol: 
1484                              if do_fit: 
1485                                  ixs_lo = max([0,max_ival-int(lookahead/4.)]) 
1486                                  ixs_hi = min([max_ix,max_ival+int(lookahead/4.)]) 
1487                                  res = fit_fn.fit(x['t'][ixs_lo:ixs_hi], 
1488                                                        x[c][ixs_lo:ixs_hi]) 
1489                                  xs_fit = res.ys_fit 
1490                                  p = res.pars_fit 
1491                                  max_tval, max_xval = res.results.peak 
1492                              else: 
1493                                  max_tval = x['t'][max_ival] 
1494                                  max_xval = x[c][max_ival] 
1495                              if verbose: 
1496                                  print "found max for %s at "%c, max_tval 
1497                              maxs_t[c].append(max_tval) 
1498                              maxs_v[c].append(max_xval) 
1499                              last_t[c][1]=max_tval 
1500                              detect_on[ci][0] = False 
1501                              detect_on[ci][1] = False 
1502                              last_type[ci]=1 
1503  ##                    else: 
1504  ##                        print "    ... ignoring b/c not detecting" 
1505          last_inc = v 
1506      return (mins_t, maxs_t, mins_v, maxs_v)

1507   
1508   


1509 -def get_extrema_from_events(gen, coords, tmin, tmax, per, pertol_frac, 
1510                              verbose=False):


1511      """Helper function for qualitative fitting of extrema using least squares. 
1512      This function returns the variable values at the extrema, unlike the 
1513      related function get_extrema (for data). 
1514   
1515      per is an estimate of the period, per <= tmax. 
1516      pertol_frac is fraction of period used as tolerance for finding extrema. 
1517      """ 
1518      evdict = gen.getEvents() 
1519      assert pertol_frac < 1 and pertol_frac > 0 
1520      assert per > 0 and per <= tmax 
1521      detect_tol = pertol_frac*per 
1522      halfper=per/2. 
1523      ms={0:'min', 1:'max'} 
1524      maxs_t = {}.fromkeys(coords) 
1525      mins_t = {}.fromkeys(coords) 
1526      maxs_v = {}.fromkeys(coords) 
1527      mins_v = {}.fromkeys(coords) 
1528      last_t = {}.fromkeys(coords) 
1529      last_type = {}.fromkeys(coords) 
1530      detect_on = [] 
1531      for c in coords: 
1532          maxs_t[c] = [] 
1533          maxs_v[c] = [] 
1534          mins_t[c] = [] 
1535          mins_v[c] = [] 
1536          last_t[c] = [-1,-1] 
1537          last_type[c] = -1 
1538          detect_on.append([True,True])   # False when outside of pertol_frac tolerance for each max and min 
1539   
1540      ev_list = [] 
1541      for ci, c in enumerate(coords): 
1542          for ei, evt in enumerate(evdict['min_ev_'+c]['t']): 
1543              ev_list.append((evt, 'min', ei, c, ci)) 
1544          for ei, evt in enumerate(evdict['max_ev_'+c]['t']): 
1545              ev_list.append((evt, 'max', ei, c, ci)) 
1546      # sort on time 
1547      ev_list.sort() 
1548   
1549      for (tval, ex_type, ei, coord, coord_ix) in ev_list: 
1550          if tval < tmin or tval > tmax: 
1551              continue 
1552          if verbose: 
1553              print "******* t = ", tval 
1554          for ci, c in enumerate(coords): 
1555              for m in [0,1]: 
1556                  if not detect_on[ci][m]: 
1557                      if tval>(last_t[c][m]+per-detect_tol): 
1558                          if not detect_on[ci][1-m] and 1-m == last_type[ci]: 
1559                              if tval>(last_t[c][1-m]+halfper-detect_tol): 
1560                                  detect_on[ci][m] = True 
1561                                  if verbose: 
1562                                      print " + %s detect (>half per of %s) for %s now True:"%(ms[m],ms[1-m],c), last_t[c][1-m], last_t[c][1-m]+halfper-detect_tol 
1563                          # The next segment allows consecutive local extrema of the 
1564                          # same type without an intermediate of the other type. 
1565                          # This is generally not desirable! 
1566  ##                        else: 
1567  ##                            detect_on[ci][m] = True 
1568  ##                            if verbose: 
1569  ##                                print " + %s detect (>per) for %s now True:"%(ms[m],c), last_t[c][m], last_t[c][m]+per-detect_tol 
1570  ##                    elif tval>(last_t[c][m]+halfper-detect_tol) and m==last_type[ci] and not detect_on[ci][1-m]: 
1571  ##                        detect_on[ci][1-m] = True 
1572  ##                        if verbose: 
1573  ##                            print " + %s detect (>half per) for %s now True:"%(ms[1-m],c), last_t[c][m], last_t[c][m]+halfper-detect_tol 
1574              do_anything = detect_on[coord_ix][0] or detect_on[coord_ix][1] 
1575              if verbose: 
1576                  print "Detecting for %s? (min=%i) (max=%i)"%(c, int(detect_on[ci][0]), int(detect_on[ci][1])) 
1577              if do_anything: 
1578                  if ex_type == 'min': 
1579                      if detect_on[coord_ix][0]: 
1580                          min_tval = tval 
1581                          if verbose: 
1582                              print "... found min for %s at "%coord, min_tval 
1583                          mins_t[coord].append(min_tval) 
1584                          mins_v[coord].append(evdict['min_ev_'+coord][ei][coord]) 
1585                          last_t[coord][0]=min_tval 
1586                          detect_on[coord_ix][0] = False 
1587                          detect_on[coord_ix][1] = False 
1588                          last_type[coord_ix]=0 
1589                  else: 
1590                      if detect_on[coord_ix][1]: 
1591                          max_tval = tval 
1592                          if verbose: 
1593                              print "... found max for %s at "%coord, max_tval 
1594                          maxs_t[coord].append(max_tval) 
1595                          maxs_v[coord].append(evdict['max_ev_'+coord][ei][coord]) 
1596                          last_t[coord][1]=max_tval 
1597                          detect_on[coord_ix][0] = False 
1598                          detect_on[coord_ix][1] = False 
1599                          last_type[coord_ix]=1 
1600      return (mins_t, maxs_t, mins_v, maxs_v)

1601   
1602   


1603 -def compare_data_from_events(gen, coords, traj, tmesh, data_mins_t, data_maxs_t, 
1604                    data_mins_v, data_maxs_v, num_expected_mins, num_expected_maxs, 
1605                    tdetect, per, pertolfrac, verbose=False):


1606      test_mins_t, test_maxs_t, test_mins_v, test_maxs_v = \ 
1607                   get_extrema_from_events(gen, coords, 
1608                                         tdetect[0], tdetect[1], 
1609                                         per, pertolfrac, verbose=verbose) 
1610      try: 
1611          num_mins = [min([len(test_mins_t[c]), len(data_mins_t[c])]) for c in coords] 
1612          num_maxs = [min([len(test_maxs_t[c]), len(data_maxs_t[c])]) for c in coords] 
1613      except TypeError: 
1614          print "Problem with mins and maxs in coords %s in generator %s"%(str(coords),gen.name) 
1615          print "Number of events found =", len(gen.getEvents()) 
1616          print per, pertolfrac, tdetect 
1617          print type(test_mins_t), type(test_maxs_t) 
1618          print type(data_mins_t), type(data_maxs_t) 
1619          raise 
1620      res_mins_t = [] 
1621      res_maxs_t = [] 
1622      res_mins_v = [] 
1623      res_maxs_v = [] 
1624      for ci,c in enumerate(coords): 
1625          nmin = num_mins[ci] 
1626          if len(data_mins_t[c]) != num_expected_mins or len(test_mins_t[c]) < num_expected_mins: 
1627              print "Wrong number of minima for %s (expected %i)"%(c, num_expected_mins) 
1628              print data_mins_t[c], test_mins_t[c] 
1629              raise RuntimeError("Wrong number of minima") 
1630          # assume 0:num_expected is good 
1631          t1 = array(data_mins_t[c])-array(test_mins_t[c])[:num_expected_mins] 
1632          v1 = data_mins_v[c]-array(test_mins_v[c])[:num_expected_mins] 
1633          res_mins_t.extend(list(t1)) 
1634          res_mins_v.extend(list(ravel(v1))) 
1635          # max 
1636          nmax = num_maxs[ci] 
1637          if len(data_maxs_t[c]) != num_expected_maxs or len(test_maxs_t[c]) < num_expected_maxs: 
1638              print "Wrong number of maxima for %s (expected %i)"%(c, num_expected_maxs) 
1639              print data_maxs_t[c], test_maxs_t[c] 
1640              raise RuntimeError("Wrong number of maxima") 
1641          # assume 0:num_expected is good 
1642          t1 = array(data_maxs_t[c])-array(test_maxs_t[c])[:num_expected_maxs] 
1643          v1 = data_maxs_v[c]-array(test_maxs_v[c])[:num_expected_maxs] 
1644          res_maxs_t.extend(list(t1)) 
1645          res_maxs_v.extend(list(ravel(v1))) 
1646      return (res_mins_t, res_mins_v, res_maxs_t, res_maxs_v)

1647
```

  


| Home | Trees | Indices | Help | PyDSTool |
| --- | --- | --- | --- | --- |

|  |  |
| --- | --- |
| Generated by Epydoc 3.0.1 on Fri May 4 15:24:22 2012 | http://epydoc.sourceforge.net |
